# Endoscopic Calcaneoplasty With Bony Debridement and Radiofrequency

**DOI:** 10.1016/j.eats.2024.102912

**Published:** 2024-02-01

**Authors:** Jun Rui Don Koh, Charles Kam King Kon, S.B.M. Darshana Chandrakumara

**Affiliations:** Changi General Hospital, Singapore

## Abstract

Haglund’s deformity refers to the enlargement of the posterosuperior tuberosity of the calcaneus, which can cause debilitating posterior heel pain and swelling. Calcaneoplasty is indicated for the treatment of Haglund’s deformity following a failure of conservative treatment. Endoscopic calcaneoplasty confers a few advantages over open surgery and has been growing in popularity as the preferred technique. This Technical Note presents an endoscopic calcaneoplasty technique with bone debridement followed by treatment with a Topaz radiofrequency device, with the patient in a supine position.

Haglund’s deformity is the bony enlargement of the posterolateral corner of the calcaneus. Ill-fitted footwear and walking can cause an exacerbation of Haglund’s syndrome, which includes retrocalcaneal bursitis and insertional Achilles tendinopathy. Conservative management is the first line of treatment and includes footwear and activity modification, physical therapy, nonsteroidal anti-inflammatory medications, and even steroid injections into the retrocalcaneal space.

Surgery is indicated for those who have failed a trial of conservative management and classically refer to open calcaneal resection or wedge osteotomy. Endoscopic calcaneoplasty was first described by van Dijk et al.^1^ in 2001 as an alternative to open surgery, with good outcomes being reported.^2^^,^^3^ Endoscopic surgery offers advantages of less operating time and reduced postoperative complications such as Achilles tendon avulsion, surgical wound issues, and postoperative stiffness and pain^4^^,^^5^ while attaining equally good outcomes as open surgery.^6^ Techniques for endoscopic calcaneoplasty have been described with variations in portals used and patient positioning.^7^, ^8^, ^9^

In this Technical Note, we describe our preferred technique, endoscopic calcaneoplasty with bone debridement followed by treatment with a Topaz radiofrequency, with the patient in a supine position (Table 1).

**Table 1:** Pearls/Pitfalls

Pearls A triangular cushion is positioned beneath the knee to flex both the hip and knee. The ankle is passively held in plantarflexion due to gravity. There are no absolute contraindications to radiofrequency application.
Pitfalls Concomitant intratendinous lesions of the Achilles tendon are not addressable by this technique. Amount of bone resection

## Arthroscopic Calcaneoplasty Technique

### Patient Preparation and Positioning

**Fig 1:**
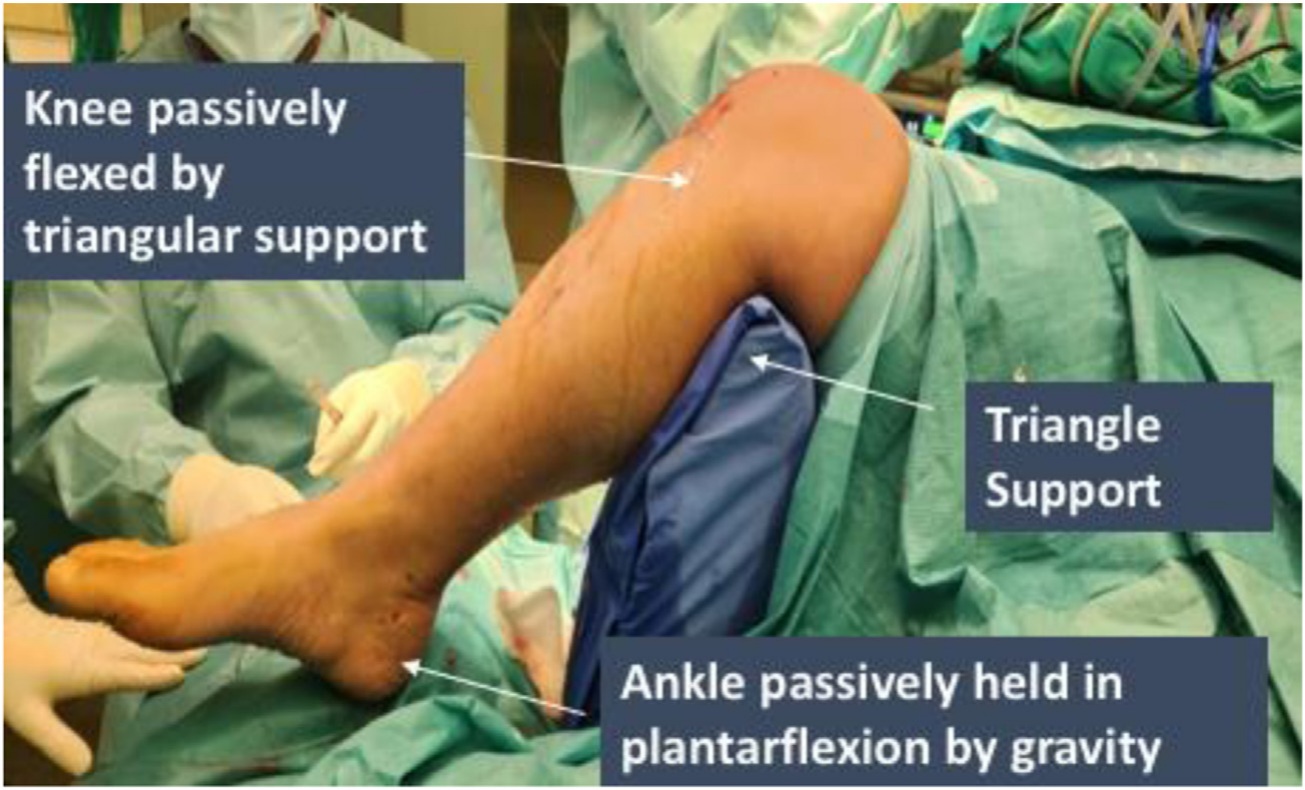
Endoscopic calcaneoplasty of the right leg. A triangle support is placed under the right knee to flex the hip and knee. The operated ankle is passively held in plantarflexion due to the assistance of gravity.

The patient is administered general or regional anesthesia and then positioned in a comfortable supine posture. To ensure optimal support, a triangular cushion is positioned beneath the knee, permitting flexion of both the hip and knee and allowing gravity to assist in passive plantarflexion of the ankle, as seen in Figure 1. Additionally, a tourniquet is applied to the limb and inflated following a meticulous cleaning and draping procedure, adhering to standard protocols.

### Portal Placement

**Fig 2:**
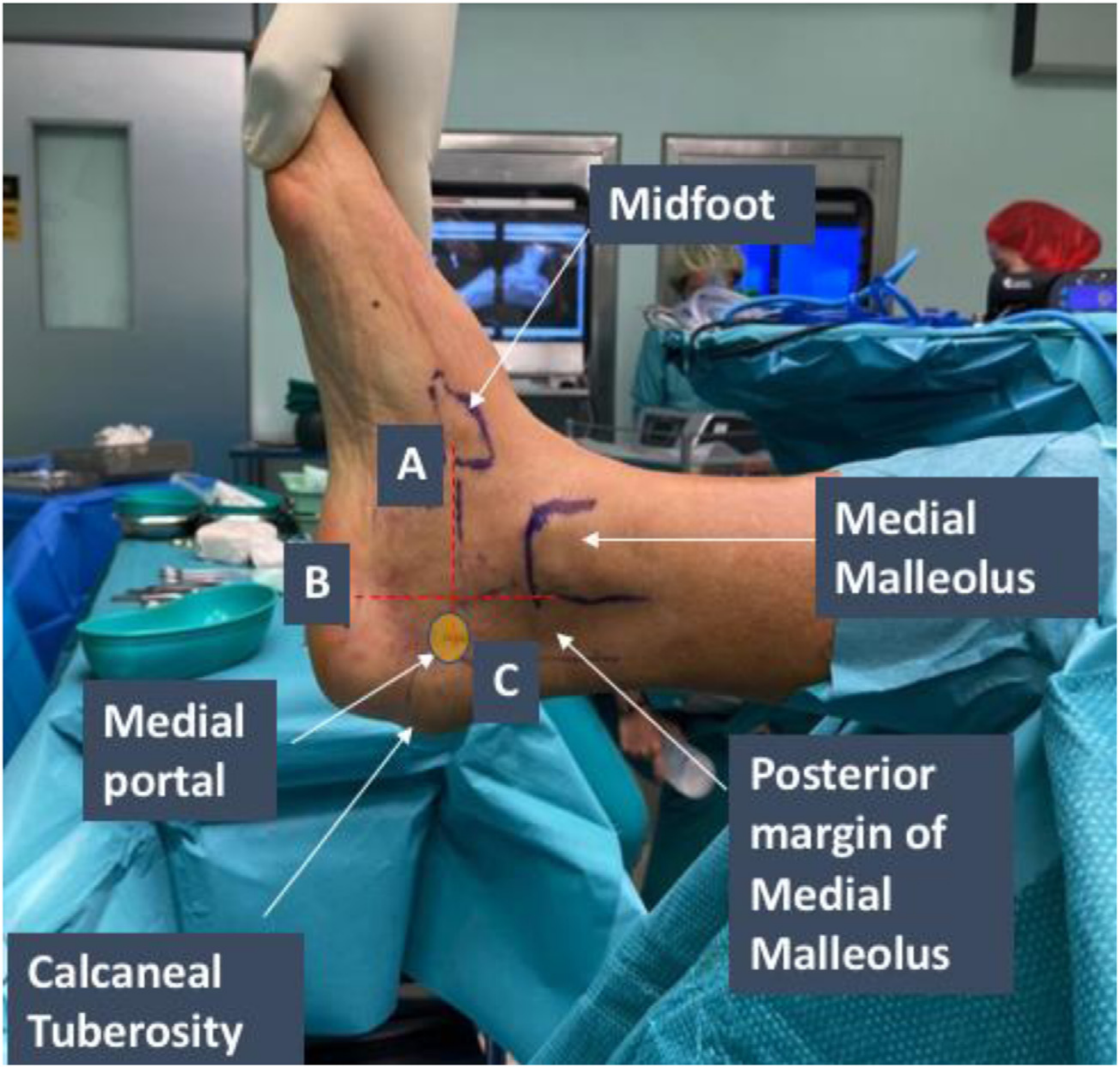
Endoscopic calcaneoplasty of the right leg. Medial portal placement is identified by drawing a plane from the midfoot to the calcaneal tuberosity (A) and a plane extending inferiorly along the posterior margin of the medial malleolus (B). The medial portal is placed just posterior to the intersection between the 2 planes (C).

Medial and lateral endoscopic portals are used for this procedure. The site of medial portal placement is first identified by drawing a plane from the midfoot to the calcaneal tuberosity and a plane extending inferiorly along the posterior margin of the medial malleolus. The medial portal is placed just posterior to the intersection drawn between the 2 planes described above. The anatomic landmarks used for siting the medial portal are displayed in Figure 2.

Following identification of the site of medial portal placement, a skin incision is first made, followed by an opening osteotomy using a 3-mm × 20-mm straight Minimally Invasive Burr (Arthrex) to increase the working area for introduction of instruments into the retrocalcaneal space. A blunt trocar is then inserted into the space and subsequently replaced with a 30-degree 4-mm arthroscope (LENS Integrated System; Smith & Nephew).

**Fig 3:**
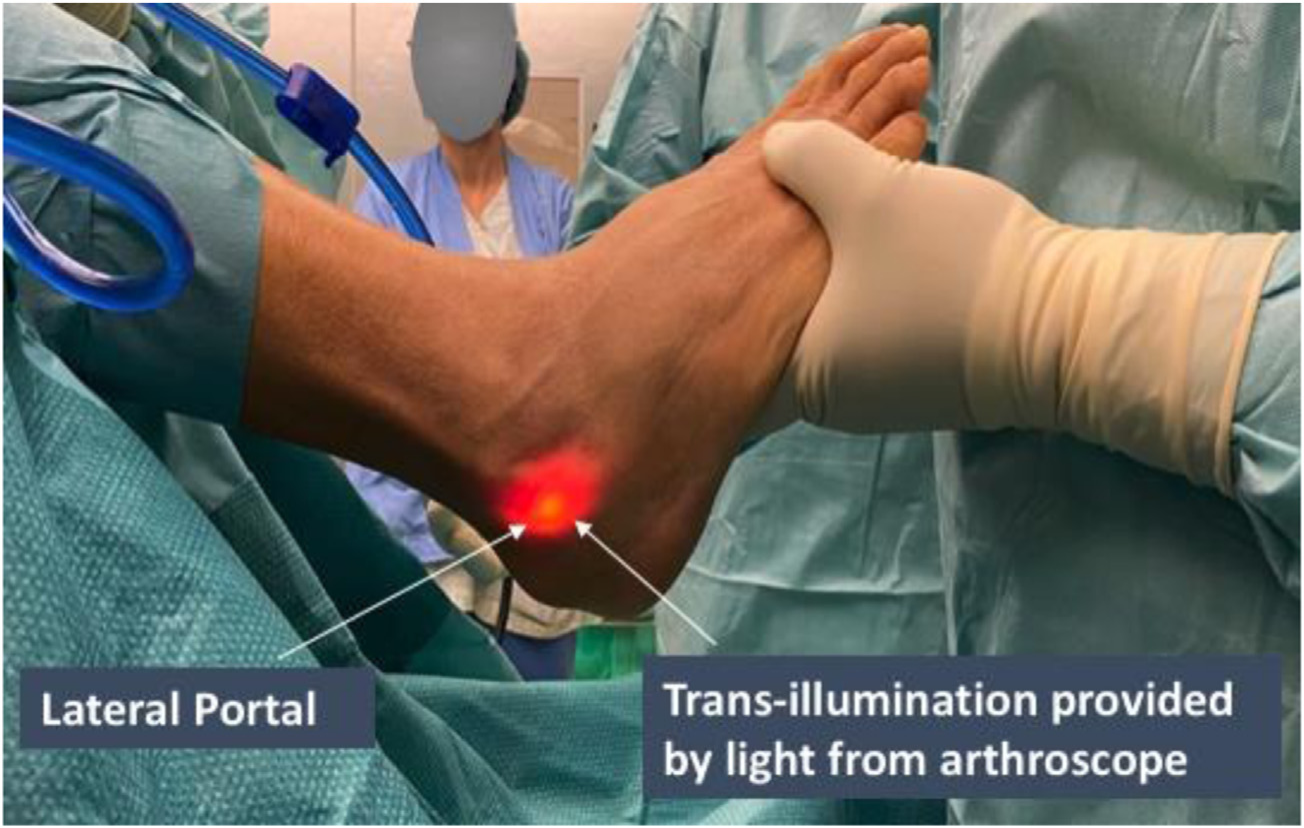
Endoscopic calcaneoplasty of the right leg. Site of lateral portal placement is identified by transillumination of the skin with light provided by the arthroscope.

The site of lateral portal placement is identified by transillumination of the skin with light from the arthroscope, seen in Figure 3. A skin incision is made again over the lateral portal entry site, followed by release of subcutaneous tissue to allow introduction of the arthroscopic shaver.

**Fig 4:**
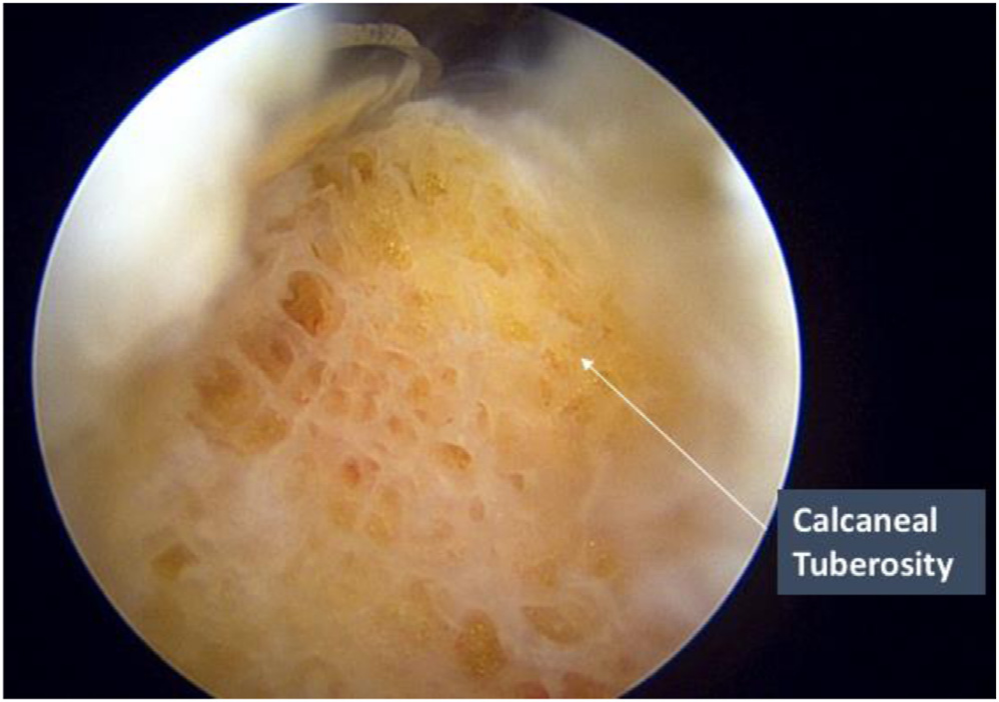
Endoscopic calcaneoplasty of the right leg. Soft tissue is cleared with use of an arthroscopic shaver or a coblation wand until the posterior calcaneal tuberosity is fully visualized.

Once the supracalcaneal space has been entered, soft tissue is systematically cleared with the help of DYONICS platinum 3.5 Blade (Smith & Nephew) and the Werewolf Flow 50 (Smith & Nephew), alternating between the two as required. Both medial and lateral portals are interchangeable as the viewing and working portals for soft tissue debridement. Care should be made to ensure the instruments point toward the viewing portal and deep to the Achilles tendon to prevent iatrogenic injury during debridement. Soft tissue should be systematically cleared proximally to distally under direct vision until the posterior calcaneal tuberosity is fully visualized (Fig 4) before proceeding further with bone resection to prevent injury to the Achilles tendon insertion.

### Bone Debridement

Bone resection is performed under direct vision using the 3.4-mm Bonecutter platinum (Smith & Nephew). The Bonecutter should be aimed toward the viewing portal and deep to the Achilles tendon. Bone resection is performed until both a smooth flat surface is achieved and the tendon Achilles attachment junction to the calcaneal tuberosity is fully visualized.

**Fig 5:**
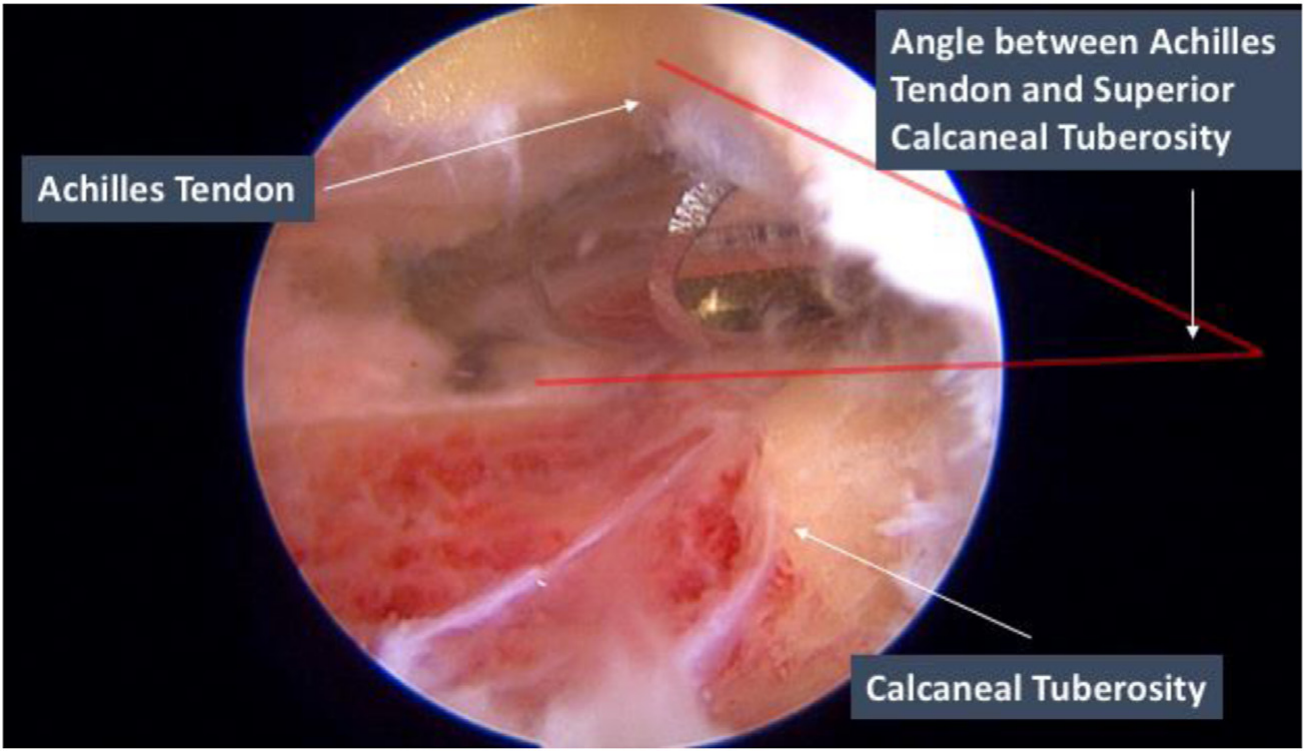
Endoscopic calcaneoplasty of the right leg. Lateral portal as the viewing portal and medial portal as the working portal. Bone resection of the calcaneal tuberosity is performed using the Bonecutter. The Bonecutter is aimed toward the viewing portal and deep to the Achilles tendon. The ankle is passively plantarflexed to relax the Achilles tendon and increase the angle between the Achilles tendon and superior calcaneal tuberosity to allow more room for bone resection and to prevent iatrogenic injury.

Bone resection should be performed distally to proximally when working in close proximity to the Achilles tendon insertion to prevent iatrogenic injury. At this time, the ankle can be further passively plantarflexed to relax the Achilles tendon and increase the angle between the Achilles tendon and superior calcaneal tuberosity to allow more room for more resection (Fig 5).

**Fig 6:**
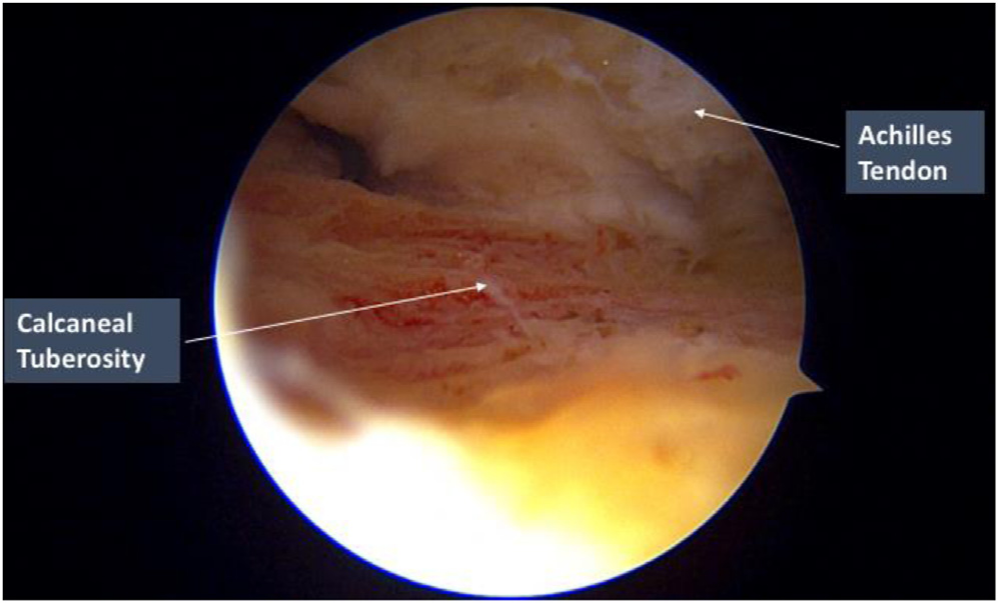
Endoscopic calcaneoplasty of the right leg. The lateral portal is the viewing portal. Bone resection is performed until a smooth, flat surface is achieved. The Achilles tendon attachment junction is also observed while passively ranging the ankle to ensure no residual impingement or contact between the tendon and any bony prominences.

The medial and lateral portals can be exchanged as the viewing and working portals to provide better working angles for bone resection of the medial and lateral halves of the posterior calcaneal tuberosity. To ensure that sufficient bone resection has been performed, the ankle can be passively ranged while observing for any impingement or contact between residual bony prominences and Achilles tendon at its attachment junction. In addition, the calcaneal tuberosity is also palpated digitally to confirm that no sharp prominent edges remain (Fig 6).

### Treatment With a Topaz Radiofrequency Device

Following completion of the endoscopic calcaneoplasty, radiofrequency therapy is applied (Topaz EZ IFS; Smith & Nephew). The patient is kept in the supine position, and the bony calcaneal tuberosity and the edges of the Achilles tendon are then palpated to identify the site of the Achilles tendon insertion. The ankle can be passively ranged to assist with identification of this anatomic structure. Radiofrequency application at the insertion site of the Achilles tendon is applied 6 times in a grid-like pattern, at intervals of 5 to 8 mm. The sites can be first marked out with use of a skin marker to ensure sufficient distance between each site. The radiofrequency is kept at the default set point of 4.

**Fig 7:**
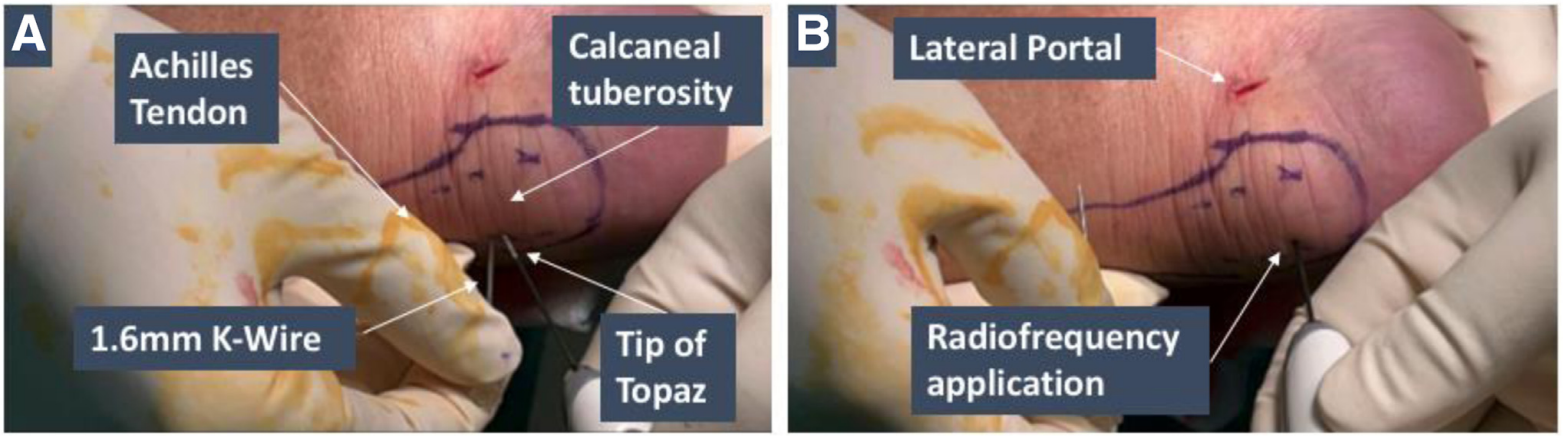
Radiofrequency application to the right leg. The Achilles tendon insertion site is identified by palpating the calcaneal tuberosity and edges of Achilles tendon. The sites of radiofrequency application are marked out in a grid-like pattern with intervals of 5 to 8 mm. A puncture in the skin is first made with a 1.6-mm K-wire (A), before introducing the tip of the Topaz (B), and the ablation function activated while simultaneously advancing the tip further into the tissue.

For each site of application, a small puncture of the skin to expose the underlying tendinous tissue is first made with use of either a 14-gauge needle or a 1.6-mm Kirschner wire, followed by introduction of the tip of the Topaz radiofrequency device. Care must be taken to ensure that only the skin layer is punctured, and the tip of the device is introduced past the dermis to ensure that the radiofrequency is applied to the tendinous tissue and to prevent burns to the superficial skin layer. The ablation function is then activated with either before advancing the tip further into the tissue to a depth of 1 mm for 0.5 seconds. The above procedure is then repeated for the rest of the remaining sites. Radiofrequency application can be performed either proximally to distally or vice versa. The above is illustrated in Figure 7.

The above surgical procedure is demonstrated in Video 1.

### Postoperative Protocol

The procedure is routinely less than a 24-hour inpatient stay, and the patient is reviewed by the physical therapist and surgical team before discharge. The patient is allowed full weightbearing and active range of motion immediately postoperatively.

## Discussion

This Technical Note describes endoscopic calcaneoplasty with bony debridement and Topaz radiofrequency application with the patient in a supine position.

While calcaneoplasty has traditionally been performed via open surgery, a rising trend in patient preferences for minimally invasive surgery supports the shift toward an endoscopic approach as the preferred practice. Good clinical outcomes after endoscopic calcaneoplasty have been previously described by various studies,^2^^,^^10^ and these outcomes were also recently shown by Yuen et al.^6^ to be comparable to outcomes after open surgery.

Endoscopic calcaneoplasty offers various advantages over open surgery. Better visualization of structures during arthroscopy allows for better identification of the portion of the calcaneal tuberosity causing impingement and therefore facilitates a more controlled and precise decompression. The extent of bone debridement was shown to be much greater if done via an open approach compared to an endoscopic approach.^11^ Over-resection in open calcaneoplasty leads to bony complications such as iatrogenic fractures, as well as postoperative complications such as stiffness and pain.^12^^,^^13^ In addition, open calcaneoplasty has also been shown to result in prolonged postoperative recovery periods.^14^

Endoscopic calcaneoplasty with the patient supine as previously described by Lui^9^ in 2016 is our preferred patient positioning of choice. The supine position confers ergonomic advantages to the surgeon, as well as reduces the positional-related surgical risks that were faced if performed in the previously described prone position.^15^

Application of Topaz radiofrequency application has also been shown to have good outcomes in treating Achilles tendinopathy.^16^^,^^17^ Topaz radiofrequency therapy in addition to calcaneoplasty can be considered for the treatment of Haglund’s syndrome, although there have been reports of complications such as tendon rupture and delayed relief, and long-term data are currently still lacking (Table 2).^18^

**Table 2:** Advantages/Disadvantages

Advantages Lower risks of complications compared to open surgery Faster postoperative recovery compared to open surgery Greater control during bony resection due to better orientation of structures Shallow learning curve for radiofrequency application
Disadvantages Risk of injury to Achilles tendon insertion during bony debridement Risk of under-resection of bone compared to open surgery Risk of Achilles tendon rupture due to inappropriate radiofrequency application

## Disclosures

The authors report no conflicts of interest in the authorship and publication of this article. Full ICMJE author disclosure forms are available for this article online, as supplementary material.

## Supplementary Data

ICMJE author disclosure forms

Video 1 Showing the surgical technique for endoscopic calcaneoplasty with bony debridement and radiofrequency.
